# Synchronization of human retinal pigment epithelial-1 cells in mitosis

**DOI:** 10.1242/jcs.247940

**Published:** 2020-09-17

**Authors:** Stacey J. Scott, Kethan S. Suvarna, Pier Paolo D'Avino

**Affiliations:** Department of Pathology, University of Cambridge, Tennis Court Road, Cambridge CB2 1QP, UK

**Keywords:** Cell division, Kinetochore, Nocodazole, Palbociclib, Synchronization

## Abstract

Human retinal pigment epithelial-1 (RPE-1) cells are increasingly being used as a model to study mitosis because they represent a non-transformed alternative to cancer cell lines, such as HeLa cervical adenocarcinoma cells. However, the lack of an efficient method to synchronize RPE-1 cells in mitosis precludes their application for large-scale biochemical and proteomics assays. Here, we report a protocol to synchronize RPE-1 cells based on sequential treatments with the Cdk4 and Cdk6 inhibitor PD 0332991 (palbociclib) and the microtubule-depolymerizing drug nocodazole. With this method, the vast majority (80–90%) of RPE-1 cells arrested at prometaphase and exited mitosis synchronously after release from nocodazole. Moreover, the cells fully recovered and re-entered the cell cycle after the palbociclib–nocodazole block. Finally, we show that this protocol could be successfully employed for the characterization of the protein–protein interaction network of the kinetochore protein Ndc80 by immunoprecipitation coupled with mass spectrometry. This synchronization method significantly expands the versatility and applicability of RPE-1 cells to the study of cell division and might be applied to other cell lines that do not respond to treatments with DNA synthesis inhibitors.

## Abstract (precis)

**Summary:** A new methodology to synchronize RPE-1 cells in mitosis for large-scale biochemical and proteomics studies.

## INTRODUCTION

Cell division is essential for growth, development and reproduction in most organisms, and errors during this process are responsible or have been implicated in several human diseases, including cancer pathologies. The process of mitosis ensures the faithful and accurate segregation of both genomic and cytoplasmic contents into two daughter cells. Mitosis progresses through a series of phases – prophase, prometaphase, metaphase, anaphase and telophase – during which the microtubule spindle is assembled in order to align and segregate the duplicated chromatids (Reber and Hyman, 2015). The centromeric regions of chromatids attach to spindle microtubules through the kinetochore, a macromolecular structure composed of a multitude of proteins and protein complexes (Cheeseman, 2014). The kinetochore is divided into two layers, the inner and outer kinetochore. The inner kinetochore comprises many CENP proteins that assemble to form the constitutive centromere-associated network (CCAN) (Cheeseman, 2014), whereas the outer kinetochore is comprised primarily of the large multi-subunit Knl1/Mis12/Ndc80 complex network (KMN network), which is recruited by the CCAN at the inner kinetochore to form strong interactions with mitotic spindle microtubules (Cheeseman et al., 2006). After all chromatids form correct bipolar attachments, the spindle assembly checkpoint (SAC) is satisfied, cyclin B is degraded, and cells can exit mitosis and segregate chromatids towards opposite poles during anaphase (Wieser and Pines, 2015). After anaphase onset, the mitotic spindle is reorganized into an array of antiparallel and interdigitating microtubules known as the central spindle that, together with the contraction of an equatorial actomyosin ring, drives the separation of the two daughter cells during cytokinesis, completing the cell division process (D'Avino et al., 2015).

A combination of genetics, biochemical and high-resolution imaging techniques is required to finely dissect the mechanics and regulation of mitotic events (Ong and Torres, 2019). Cultured human cells have proven a very useful model for the study of mitosis, but most of the cell lines employed in the field originated from tumors and thus often have alterations in the main genes and signaling pathways that regulate cell growth, the cell cycle and mitosis. For example, HeLa cervical adenocarcinoma cells (Jones et al., 1971) are widely used in mitotic studies because they are easy to image, grow in large quantities and can be easily synchronized at different mitotic stages using a thymidine–nocodazole block-and-release method. However, HeLa cells are transformed by the human papilloma virus, which silences the crucial tumor suppressor p53 (Scheffner et al., 1991; Yee et al., 1985). Moreover, these cells are genetically unstable and near-tetraploid, which makes the use of gene-editing techniques laborious and time-consuming because of the presence of multiple gene copies. For these reasons, the diploid, non-transformed, hTERT-immortalized, retinal pigment epithelial-1 (RPE-1) cell line (Bodnar et al., 1998) is emerging as a very valid alternative to HeLa cells for the study of mitosis. However, an effective method to synchronize these cells in mitosis is still lacking, which precludes their use for large-scale biochemical and molecular studies aimed at dissecting the functions, regulation and interactions of mitotic proteins. Here we describe a protocol, based on combined sequential treatments with the Cdk4 and Cdk6 inhibitor PD 0332991 (palbociclib) (Fry et al., 2004) and the microtubule-depolymerizing drug nocodazole (Sentein, 1977), that can be used to efficiently synchronize RPE-1 cells in mitosis. We also demonstrate that this synchronization method can be successfully employed for the characterization of the protein–protein interaction network (interactome) of the kinetochore protein Ndc80 by immunoprecipitation coupled with mass spectrometry.

## RESULTS

### Synchronization of RPE-1 cells using a palbociclib–nocodazole block-and-release method

**Fig. 1.**
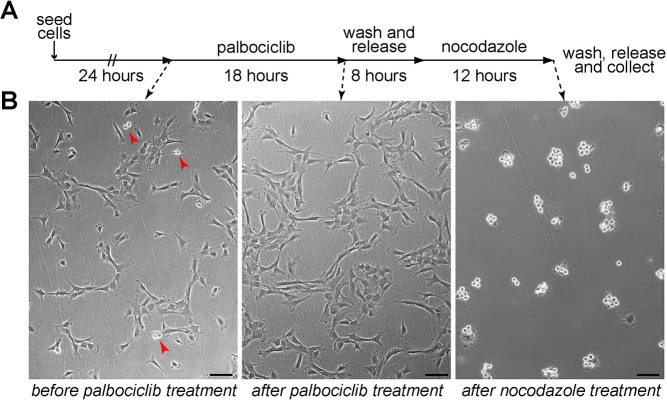
**RPE-1 cells can be synchronized in mitosis using a palbociclib–nocodazole block.** (A) Schematic diagram of the synchronization protocol. (B) Microscopy images of RPE-1 cells before and after palbociclib treatment (left and middle panels), and after nocodazole incubation (right panel). The red arrowheads in the left panel mark dividing cells. Note that the vast majority of cells are round dividing cells after nocodazole treatment. Scale bars: 100 µm.

Our initial attempts to synchronize RPE-1 and other p53 wild-type cells in mitosis using DNA synthesis inhibitors, like thymidine or aphidicolin, failed because these drugs presumably triggered a DNA damage response and consequent p53 activation and cell cycle arrest (data not shown). To overcome this problem, we decided to instead employ the Cdk4 and Cdk6 inhibitor palbociclib, which blocks cells in the G1 phase of the cell cycle (Fry et al., 2004) and has been shown not to cause DNA damage in a variety of cell types, including non-transformed epithelial cells (Dean et al., 2012; DiRocco et al., 2014; Hu et al., 2016; Roberts et al., 2012). RPE-1 cells were treated with palbociclib for 18 h, released for 8 h and then incubated with nocodazole for 12 h. After nocodazole treatment, cells were extensively washed, released in fresh medium, and collected at different time intervals (Fig. 1A; see also Materials and Methods). We observed almost no rounded-up mitotic cells after palbociclib treatment, indicating that the cells arrested in G1 (Fig. 1B). By contrast, the vast majority of cells (80–90%) after incubation with nocodazole appeared to be in mitosis (Fig. 1B).

**Fig. 2.**
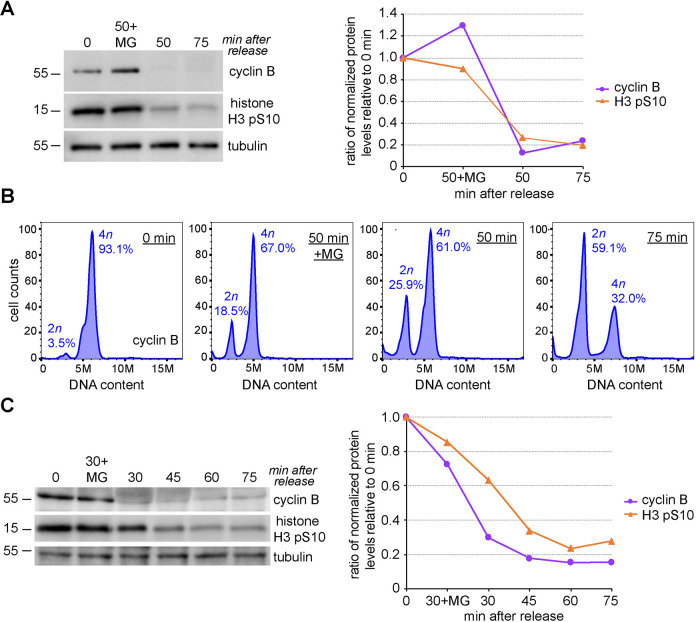
**RPE-1 cells exit mitosis synchronously after release from the nocodazole block.** (A) Analysis of protein expression in RPE-1 cells released from nocodazole. Proteins were extracted from RPE-1 cells at the times indicated at the top, separated by SDS-PAGE, and analyzed by western blotting to identify the proteins indicated to the right. The numbers on the left indicate the sizes of the molecular mass markers. The graph on the right shows the quantification of protein levels for the representative experiment on the left, normalized to tubulin and relative to levels at time 0. (B) Flow cytometry profiles of cells released from the nocodazole block at the same time points as in A and stained with propidium iodide (PI) to analyze DNA content. (C) Proteins were extracted from RPE-1 cells at the times indicated at the top, separated by SDS-PAGE, and analyzed by western blotting to identify the proteins indicated to the right. The numbers on the left indicate the sizes of the molecular mass markers. The graph on the right shows the quantification of protein levels for the representative experiment on the left, normalized to tubulin and relative to levels at time 0. Data presented in A and C are representative of three independent experiments.

We next analyzed RPE-1 cells collected at 0, 50 and 75 min after nocodazole release by both western blot and flow cytometry analysis to identify their mitotic stages (Fig. 2). We also included a sample of cells released for 50 min in medium containing the proteasome inhibitor MG132, which should prevent cyclin B degradation and mitotic exit. The levels of both cyclin B and the mitotic marker histone H3 phosphorylated at S10 (H3 pS10) dropped by more than 70–80% after 50 min (Fig. 2A), indicating that the SAC had been satisfied and most cells had exited mitosis. By contrast, the levels of both mitotic markers were unaltered or even increased in cells released for 50 min in medium with MG132 (Fig. 2A). Flow cytometry profiles indicated that more than 90% of the cells at the 0 min time-point had 4*n* DNA content and that this percentage gradually decreased after 50 and 75 min. This decrease in 4*n* cells was paralleled by an increase in the number of 2*n* G1 cells, which were the majority in the 75 min sample (Fig. 2B). Notably, the <2*n* cell population, which reflects cell death, was very low, less than 5% in all samples (0.6% in *t*=0 min; 2.5% in *t*=50 min+MG; 4.5% in *t*=50 min; and 4.1% in *t*=75 min), indicating that sequential treatments with palbociclib and nocodazole do not significantly affect cell viability.

We further refined our western blot analysis in samples collected at more frequent intervals (30, 45, 60 and 75 min) after nocodazole release (Fig. 2C). Cyclin B levels dropped by 70% after just 30 min, while a much more modest decrease of less than 40% was observed for histone H3 pS10 (Fig. 2C). By contrast, the levels of both markers only marginally decreased in cells released for 30 min in the presence of MG132 (Fig. 2C). Cyclin B and histone H3 pS10 levels gradually decreased in the 45, 60 and 75 min samples to 20% or less compared to those at the 0 time point (Fig. 2C).

Together, these results indicate that most RPE-1 cells are in anaphase 30 min after release from nocodazole and that the majority of cells have completed mitosis and are in G1 after 60–75 min.

### RPE-1 cells recover after the palbociclib–nocodazole block

**Fig. 3.**
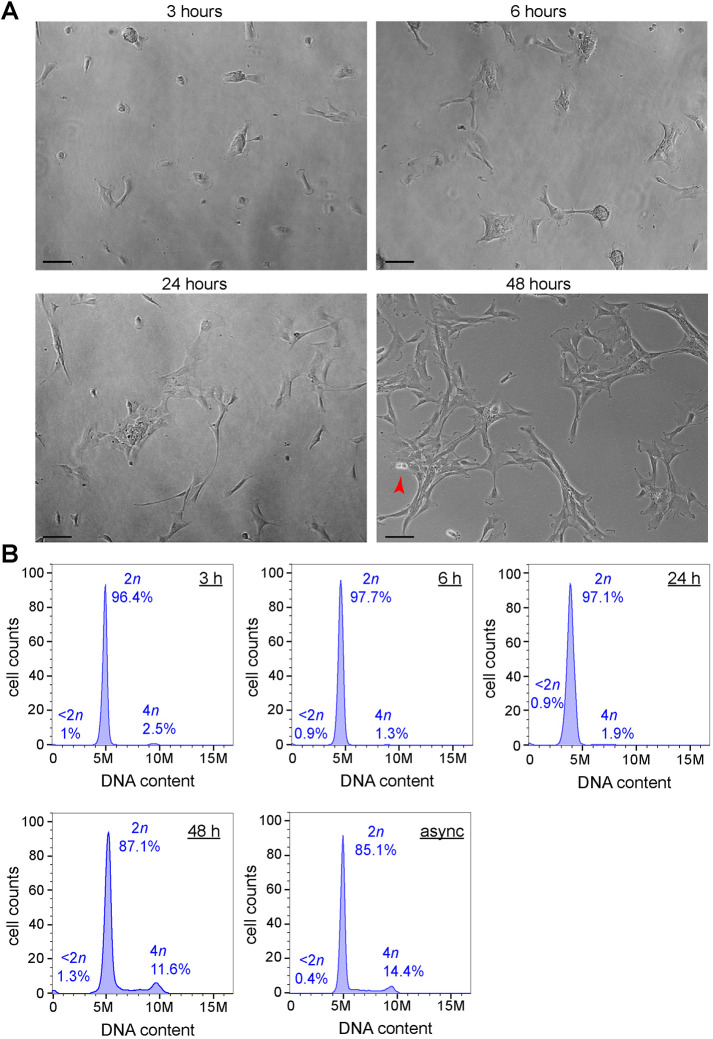
**RPE-1 cells fully recover after release from the nocodazole block.** (A) Microscopy images of RPE-1 cells taken at the indicated time points after nocadozle release. The red arrowhead in the 48 h panel marks a dividing cell in cytokinesis. Scale bars: 100 µm. (B) Flow cytometry profiles of cells released from the nocodazole block at the indicated time points and stained with propidium iodide (PI) to analyze DNA content. The profile of an asynchronous (async) cell population is also shown.

To understand whether palbociclib–nocodazole synchronization had any effect on the viability of RPE-1 cells and their ability to re-enter the cell cycle, we monitored the survival and behavior of RPE-1 cells at different time points up to 48 h after release from the palbociclib–nocodazole block. We found that RPE-1 cells started to re-attach 3 h after release from nocodazole and re-acquired their normal morphology after 24 h, and that dividing cells could be observed after 48 h (Fig. 3A). Flow cytometry profiles indicated that almost all cells were diploid after 3 h and then they slowly re-entered the cell cycle to reacquire the normal profile of an unsynchronized cell population after 48 h (Fig. 3B). Importantly, the percentage of the cell population with less that 2*n* DNA content, which reflects cell death, was consistently very low (≤1.5%). These results indicate that the vast majority of RPE-1 cells fully recover after the release from nocodazole and can successfully begin a new cell cycle.

### Characterization of the Ndc80 interactome using the palbociclib–nocodazole block-and-release method

**Fig. 4.**
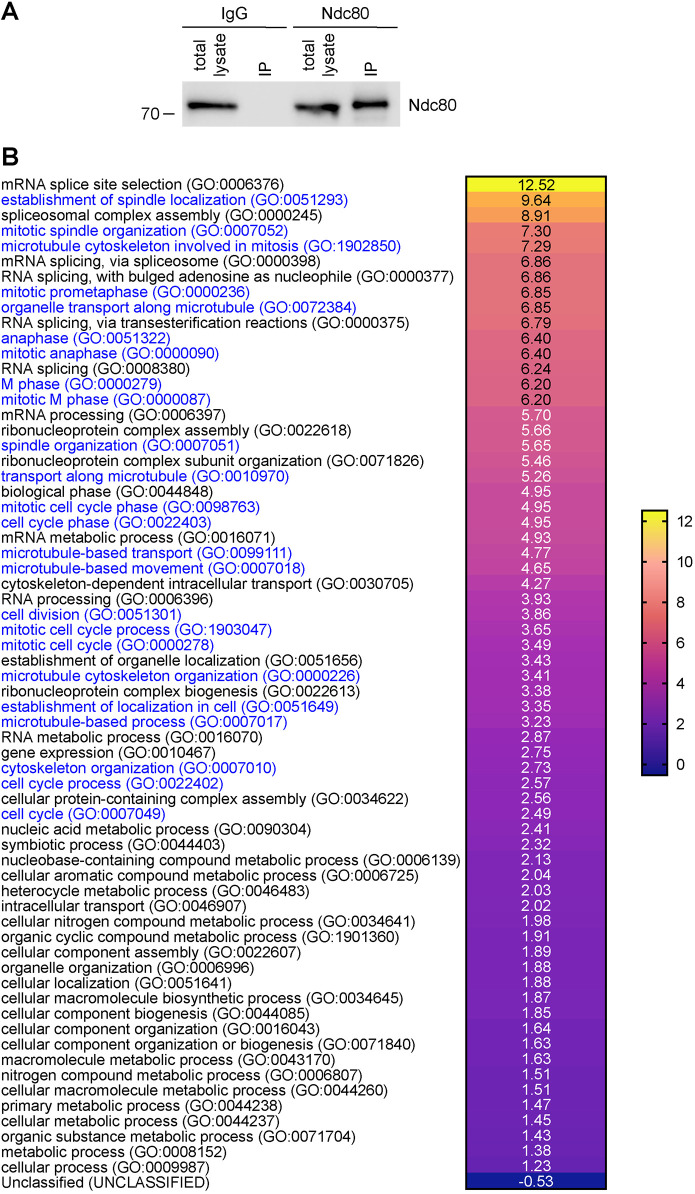
**Characterization of the Ndc80 interactome in metaphase RPE-1 cells.** (A) Protein extracts were used in IP assays using either Ndc80 or IgG, and then extracts and pull-downs were analyzed by western blotting to detect Ndc80. The numbers on the left indicate the sizes of the molecular mass marker. (B) Heat map showing the GO annotation enrichment profile of the Ndc80 interactome analyzed using PANTHER under the category GO biological process. Overrepresented GO terms are shown in different color shades according to their fold enrichment, as indicated in the color scale bar at the right; actual fold enrichment values are shown within the heat map. GO terms for processes involved in cell cycle, mitosis and microtubule dynamics are in blue. Only results for Bonferroni-corrected for *P*<0.05 were considered (see Table S2).

To demonstrate the applicability of our synchronization protocol, we decided to characterize the interactome of the outer kinetochore component Ndc80 in RPE-1 cells synchronized at metaphase. We used our palbociclib–nocodazole block-and-release method to synchronize and collect RPE-1 cells 30 min after release from nocodazole in medium containing MG132. Protein extracts from these cells were used in immunoprecipitation (IP) experiments with antibodies against the outer kinetochore component Ndc80 or general mouse IgG as control, and then bait and prey proteins were identified by mass spectrometry (Fig. 4). After eliminating non-specific preys (see Materials and Methods), we identified 380 Ndc80-specifc interactors (Table S1), which included all the four components of the Ndc80 complex (Ndc80, Nuf2, Spc24 and Spc25), as well as many other proteins known to be involved in spindle assembly and chromosome attachment and alignment (Table 1 and Table S1). As expected, the Gene Ontology (GO) enrichment profile of the Ndc80 interactome indicated a strong enrichment in proteins involved in mitosis, cell cycle, spindle assembly and microtubule dynamics (Fig. 4B; Table S2), which indicates that our synchronization method can be successfully employed in large-scale proteomic studies.

**Table 1.**
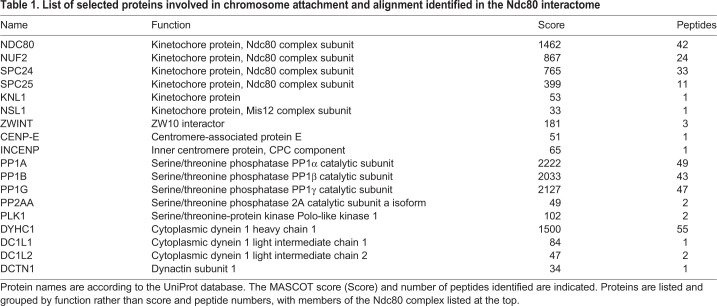
**List of selected proteins involved in chromosome attachment and alignment identified in the Ndc80 interactome**

## DISCUSSION

Our results demonstrate that combined and sequential treatments with the Cdk4 and Cdk6 inhibitor palbociclib and the microtubule-depolymerizing drug nocodazole can be successfully used to synchronize RPE-1 cells at different mitotic stages. We believe that this new technique will significantly expand the versatility and applicability of RPE-1 cells to the study of cell division, and our preliminary results indicate that this protocol can also be applied to other cell lines that have a functional p53 response or do not respond to treatments with DNA synthesis inhibitors (data not shown).

The characterization of the Ndc80 interactome in RPE-1 cells presented in this study (Fig. 4) supports the validity of our method for the large-scale purification of synchronized RPE-1 cells to use in biochemical and proteomics studies. Possible applications include not only the identification of protein–protein interaction networks, but also the characterization of post-translational modifications of the mitotic proteome. Interestingly, we found a significant enrichment in our Ndc80 interactome of proteins involved in RNA processing and splicing (Fig. 3B; Table S2). Although unexpected, this finding is consistent with the identification of the splicing factors Sf3A2 and Prp31 as Ndc80 interactors (Pellacani et al., 2018). These results, together with the evidence that splicing factors and RNA-binding proteins were also identified as cohesin interactors (Kim et al., 2019), suggest that these proteins, and possibly RNA, may play some role in mitosis; a hypothesis that deserves future investigations.

Finally, it is important to note that, although our results and data from other studies indicate that mitotic spindle assembly is not impaired after nocodazole release and the SAC is satisfied, an increase in the frequency of merotelic attachments (which are not sensed by the SAC) and chromosome mis-segregation have been reported in RPE-1 and other cell lines (Cimini et al., 2001; Worrall et al., 2018). This should be taken into consideration when analyzing the results of synchronization experiments using our method, as well as any other nocodazole-based protocol.

## MATERIALS AND METHODS

### Cell culture, synchronization and imaging

hTERT-RPE-1 cells were obtained from ATTC, quarantined and tested for mycoplasma infection. RPE-1 cells were cultured in DMEM F-12+ Glutamax medium (GIBCO) supplemented with 10% fetal bovine serum (Sigma-Aldrich), 1% penicillin-streptomycin (Thermo Fisher) and 0.01 mg ml^−1^ hygromycin B (Thermo Fisher) at 37°C and 5% CO_2_.

For synchronization in mitosis, RPE-1 cells were initially seeded at 17% confluence as this ensured that cells were sub-confluent by the time of nocodazole addition (see below), which we found to be crucial for successful synchronization. After 24 h, palbociclib (PD 0332991, Selleckchem) was added to the medium at a final concentration of 1 μM and cells were incubated for further 18 h. After three washes with phosphate-buffered saline (PBS plus CaCl_2_ and MgCl_2_) to remove the drug, cells were cultured for 8 h in fresh complete medium before adding 50 ng ml^−1^ nocodazole (Sigma-Aldrich). After 12 h, cells were harvested by mitotic shake-off, centrifuged at 1000 ***g*** for 3 min, washed five times with large volumes of PBS plus CaCl_2_ and MgCl_2_, and released in fresh complete medium with or without 10 μM MG132 (Sigma-Aldrich) for the appropriate times before collection.

Cells were imaged using a Nikon Eclipse TS100F inverted microscope equipped with a Nikon DS-Fi1 digital color camera with a 5 megapixel sensor.

### Western blot analysis

Cells were centrifuged (5000 ***g*** for 3 min), resuspended in PBS and then an equal volume of 2× Laemmli buffer was added. Samples were then boiled for 10 min and stored at −20°C. Proteins were separated by SDS PAGE and then transferred onto PVDF membrane (Immobilon-P, Millipore) at 15 V for 1 h. Membranes were blocked overnight at 4°C in PBS +0.1% (v/v) Tween 20 (PBST) with 5% (w/v) dry milk powder. After blocking, membranes were washed once with PBST and then incubated with the appropriate primary antibody diluted in PBST plus 3% (w/v) BSA (Sigma-Aldrich) for 2 h at room temperature. Membranes were washed three times for 5 min each time in PBST and then incubated with HRP-conjugated secondary antibodies in PBST plus 1% BSA for 1 h at room temperature. After a further three 5 min washes in PBST, the signals were detected using the ECL West Pico substrate (ThermoFisher) and chemiluminescent signals were acquired below saturation levels using a G:BOX Chemi XRQ (Syngene) and quantified using Fiji (Schindelin et al., 2012).

### Antibodies

The following antibodies and dilutions for western blot were used in this study: rabbit polyclonal anti-β-tubulin (Abcam, ab6046; dilution 1:5000), mouse monoclonal anti-cyclin B1 (Santa Cruz Biotechnology, clone GNS1, sc-245; dilution for 1:2000) and rabbit polyclonal anti-phospho-histone H3 pS10 (Merck, 06-570; dilution 1:10,000).

### Flow cytometry

RPE-1 cells were centrifuged at 1000 ***g*** for 3 min, the supernatant removed, and cell pellets resuspended in 5 ml of PBS. Then, 2 ml of ice-cold 70% ethanol was added drop-wise to the pellet while vortexing, and cells were maintained at −20°C for a minimum of 2 h. After centrifuging at 800 ***g*** for 5 min at 4°C, the supernatant was removed and cell pellets were washed twice with PBS before resuspending in 0.2–0.5 ml of propidium iodide (PI)/RNase staining buffer (BD Pharmigen) followed by incubation at room temperature for 15 min in the dark. Stained cells were then stored at 4°C for a minimum of 24 h, strained through a 35 µm nylon mesh, and loaded onto a CytoFLEX S (Beckman Coulter) instrument. Cytexpert software (Beckman Coulter) was used for data acquisition, a 561 nm laser with a 610/20 filter was used for detection, and 10,000 single-cell events were recorded for each sample. Annotation of data was performed manually using FlowJo software.

### Immunoprecipitation

100 µl of Dynabeads Protein A (ThermoFisher) were prepared for immunoprecipitation (IP) by washing three times with PBS followed by incubation with either 10 µg of mouse monoclonal anti-Ndc80/Hec1 antibody (Santa Cruz Biotechnology, sc-515550) or 10 µg of mouse IgG (Sigma-Aldrich) in 200 µl of PBS plus 0.1% (v/v) NP40 on a rotating wheel at 4°C overnight.

Six 175 cm^2^ flasks of RPE-1 cells were seeded at 17% confluence (∼9×10^6^ cells per flask) and synchronized in prometaphase, as described above. Cells were released from nocodazole in fresh medium containing 10 μM MG132 (Sigma-Aldrich), collected after 30 min, washed in PBS, centrifuged (1000 ***g*** for 5 min), and the cell pellet stored at −80°C. Cell pellets were thawed by directly resuspending in 2 ml of extraction buffer [EB; 50 mM HEPES pH 7, 100 mM KAc, 50 mM KCl, 2 mM MgCl_2,_ 1 mM EGTA, 0.1% (v/v) NP-40, 1 mM DTT, 5% (v/v) glycerol and Roche Complete protease inhibitors] and homogenized using a high-performance disperser (Fisher). The cell lysate was treated with 200 µg/ml of DNase I (New England BioLabs) for 10 min at 37°C followed by 10 min at room temperature and then clarified by centrifugation at 1500 ***g*** for 15 min at 4°C. After centrifugation, the supernatant was split into two and each sample was added to the Dynabeads Protein A pre-incubated with either the Ndc80 antibody or mouse IgG (see above) on a rotating wheel at 4°C for 4 h. Beads were then washed four times using a magnetic stand in 5 ml of EB for 5 min on a rotating wheel at 4°C, transferred to a new tube and washed one more time with 5 ml of PBS. After removing as much liquid as possible, beads were stored at −80°C before being analyzed by liquid chromatography coupled with tandem mass spectrometry (LC-MS/MS; see section below).

### Mass spectrometry analyses

For the analysis of IP samples, beads were digested with trypsin and processed as previously described (McKenzie et al., 2016). Raw MS/MS data were analyzed using the MASCOT search engine (Matrix Science). Peptides were searched against the UniProt human sequence database, and the following search parameters were employed: enzyme specificity was set to trypsin, a maximum of two missed cleavages were allowed, carbamidomethylation (Cys) was set as a fixed modification, whereas oxidation (Met), phosphorylation (Ser, Thr and Tyr) and ubiquitylation (Lys) were considered as variable modifications. Peptide and MS/MS tolerances were set to 25 parts per million (ppm) and 0.8 daltons (Da). Peptides with MASCOT Score exceeding the threshold value corresponding to <5% false positive rate calculated by the MASCOT procedure, and with the MASCOT score above 30 were considered to be positive.

#### Computational and statistical analyses

We used in-house written Perl scripts (available upon request) to compare the MASCOT data from the Ndc80 and IgG IP experiments in order to eliminate non-specific hits. Prey hits either absent from the IgG control dataset or having a ≥5-fold increase in both MASCOT score and peptide numbers compared to the IgG negative control were classed as being specific. Additional common contaminants, such as keratins and hemoglobin, were eliminated manually.

GO enrichment analysis was performed using PANTHER (Mi et al., 2017). Prism8 (GraphPad) and Excel (Microsoft) were used for statistical analyses and to prepare graphs.

## Supplementary Material

Supplementary information

Reviewer comments
