# Supplementary material for: Synchronization of human retinal pigment epithelial-1 cells in mitosis
Source: J Cell Sci. 2020 Sep 17;133(18):jcs247940. doi: 10.1242/jcs.247940 (PMC7520456; doi:10.1242/jcs.247940)
Supplement: Supplementary information [file joces-133-247940-s1.pdf]

Table S1. Excel file listing the proteins identified by MS from the Ndc80 IP of RPE-1 cells synchronized in metaphase. Non-specific binding proteins were removed by filtering the Ndc80 dataset against the IgG dataset (see Materials and Methods).

Click here to Download Table S1

Table S2. Excel file showing the results of the GO enrichment analyses from the MS data of the Ndc80 IP shown in Table S1.

Click here to Download Table S2

Journal of Cell Science • Supplementary information
