# Supplementary material for: Synchronization of human retinal pigment epithelial-1 cells in mitosis
Source: J Cell Sci. 2020 Sep 17;133(18):jcs247940. doi: 10.1242/jcs.247940 (PMC7520456; doi:10.1242/jcs.247940)
Supplement: Reviewer comments [file joces-133-247940_review_history.pdf]

Synchronization of human retinal pigment epithelial-1 cells in mitosis

Stacey J. Scott, Kethan S. Suvarna and Pier Paolo D'Avino
DOI: 10.1242/jcs.247940

Editor: John Heath

Review timeline

Original submission:	22 April 2020
Editorial decision:	29 May 2020
First revision received:	17 July 2020
Accepted:	18 August 2020

Original submission

First decision letter

MS ID#: JOCES/2020/247940

MS TITLE: Synchronization of human retinal pigment ephitilial-1 (RPE-1) cells in mitosis

AUTHORS: Stacey J Scott, Kethan Suvarna, and Pier Paolo D'Avino
ARTICLE TYPE: Tools and Resources

We have now reached a decision on the above manuscript.

To see the reviewers' reports and a copy of this decision letter, please go to: https://submit-jcs.biologists.org and click on the 'Manuscripts with Decisions' queue in the Author Area. (Corresponding author only has access to reviews.)

As you will see, the reviewers gave favourable reports but raised some points that will require amendments to your manuscript. I hope that you will be able to carry these out, because I would like to be able to accept your paper.

We are aware that you may currently be unable to access the lab to undertake experimental revisions. If it would be helpful, we encourage you to contact us to discuss your revision in greater detail. Please send us a point-by-point response indicating where you are able to address concerns raised (either experimentally or by changes to the text) and where you will not be able to do so within the normal timeframe of a revision. We will then provide further guidance. Please also note that we are happy to extend revision timeframes as necessary.

Please ensure that you clearly highlight all changes made in the revised manuscript. Please avoid using 'Tracked changes' in Word files as these are lost in PDF conversion.

I should be grateful if you would also provide a point-by-point response detailing how you have dealt with the points raised by the reviewers in the 'Response to Reviewers' box. Please attend to all of the reviewers' comments. If you do not agree with any of their criticisms or suggestions please explain clearly why this is so.

© 2020. Published by The Company of Biologists under the terms of the Creative Commons Attribution License (https://creativecommons.org/licenses/by/4.0/).

Reviewer 1

Advance summary and potential significance to field

This paper presents a very focused demonstration of a method that allows the preparation of populations of RPE-1 cells synchronized by enhanced mitotic arrest. The authors present the convincing argument that appropriate cell cycle analytic methods are required for the increasingly important RPE-1 cell, a model karyotypically normal, untransformed epithelial cell type. A serial G1 and mitotic block, accomplished with palbociclib and nocodazole, respectively, resulted in populations with 80-90% of cells arrested in a prometaphase-like state, which proceeded synchronously to exit mitosis upon removal of nocodazole. The utility of the mitotically arrested population was demonstrated by immunoprecipitation of Ndc80 and associated proteins from extracts prepared from synchronized cells.

In summary, this is a highly focused report describing a novel method to produce populations of nearly pure mitotic RPE-1 cells. As RPE-1 and its derivatives are increasingly important models for cell biology, it is likely that this method will be of significant utility in the field.

Comments for the author

Overall, this is a reasonably well documented description of a method that is likely to be of significant utility to labs using RPE-1 cells and derivatives to study mitosis and possibly cell cycle events. A general lack of quantitative information is seen as a limit to the utility of the method as presented. As well, the authors focused on the mitotic population and did not develop the utility of the method as a general tool for cell cycle studies. Nonetheless, the method seems sound and should be a substantive contribution to the establishment of robust methods to study the cell biology of RPE-1 cells.

Specific comments are below:

1. While the method is clear in terms of cell manipulations, culture conditions and drug concentrations, the technique begins by splitting the cells 1/6. It would be very useful to know what cell density this corresponds to to allow quantitative planning of synchrony experiments. Similarly for the preparation of extract for immunoprecipitation – how many cells is 5 x 175 cm^2? Was the protein concentration of the extract determined.
2. The authors show clearly that cells are arrested in a prometaphase-like state and removal of nocodazole results in prompt exit from mitosis. How does the population behave in the subsequent cell cycle? Is this a method that is applicable to general cell cycle studies? The authors should comment on this.
3. Ndc80 IP analysis reveals an unexpected enrichment in RNA splicing components. Recent results have shown mitotic roles for such proteins, e.g. Kim et al J Biol Chem. 2019 May 31;294(22):8760-8772 (cohesin associated), Pellacani et al Elife. 2018 Nov 26;7. pii: e40325. doi: 10.7554/eLife.40325 (Ndc80 associated).

Since this is a pronounced outcome of the IP experiment, perhaps a bit more discussion of recent findings is in order.

Reviewer 2

Advance summary and potential significance to field

This manuscript does not make a conceptual advance but is rather intended as a significant advance in methodology. It is true that RPE-1 cells have become a widely used model for studying the cell cycle and mitosis in particular, mainly through microscopy. Efficient methods of cell cycle synchronisation are of value in order to use this cell line for biochemical analysis. While some have claimed ability to synchronize these cells at G1/S with thymidine, this may be inefficient. Other methods reported for RPE-1 cells are use of a CDK1 inhibitor (RO-3306) to block cells at the G2/M border before release into mitosis, and trapping mitotic cells with nocodazole without prior synchronisation, although this latter method obviously results in fewer mitotic cells. The present method of presynchronization in G1/S with the CDK4/6 PD 0332991 (palbociclib) followed by

© 2020. Published by The Company of Biologists under the terms of the Creative Commons Attribution License (https://creativecommons.org/licenses/by/4.0/).

release and capture in mitosis with nocodazole is an addition to this methodology, albeit a minor advance in itself.

A concern (that is often neglected) with any drug-induced synchronisation method is the validity of the synchronised cells as a model for normal cell cycle states. Inevitably, synchronisation involves the induction of a stress response in the cells, which is why they halt cell cycle progression. It is important to know (1) are there persistent consequences for the cell after the drug treatment, (2) do the cells recover from the synchronisation to resume normal cell cycle processes. For instance, it is very likely that inhibition of DNA replication by thymidine synchronisation (and CDK4/6 inhibition?) causes replication stress, the effects of which may persist into a subsequent mitosis. In addition, it is clear that nocodazole arrest of cells in mitosis triggers a stress response that can even lead to cell death, depending on the duration of the arrest.
Importantly, recovery from nocodazole arrest through repolymerization of microtubules and formation of the mitotic spindle is often inefficient, leading to defects in chromosome segregation and downstream consequences, including aneuploidy and interphase stress responses. It is therefore important to fully characterise both the stress response to CDK4/6 inhibitor synchronisation followed by nocodazole synchronisation, and to demonstrate that cells thus synchronised perform a (relatively) normal mitosis.

My view is that this work would be better published as part of a more comprehensive paper on the NDC80 complex, including conceptual advances, or as a well characterised study comparing synchronisation methods to demonstrate the benefits and limitations of each method.

Comments for the author

To demonstrate that CDK4/6 inhibitor synchronisation followed by nocodazole synchronisation is a superior method of collecting cells to study mitosis, I suggest that the authors characterise the levels of key indicators of stress responses following release from each of these treatment, including DNA damage responses that would indicate whether or not the cells have replication stress or activation of mitotic stress responses.
Analysis of gamma H2AX levels would be a good place to start, preferably by microscopy but also by western blotting. The levels of cell death induced by the method will also be important to check. From the flow cytometry profiles shown, it does look like some cells with sub-2N DNA content are generated, which probably indicates apoptosis or possibly chromosome missegregation. At a low level, this might be acceptable, but it calls into question whether the synchronised cells truely have a normal mitosis and the stress response could affect mitotic events.

Secondly, it seems important to confirm that the synchronised cells perform mitotic correctly and on schedule in order to demonstrate that this is a good or improved technique for studying mitosis. This needs to be done by microscopy, preferably live cell imaging.

First revision

Author response to reviewers' comments

Reviewer 1
Advance Summary and Potential Significance to Field
This paper presents a very focused demonstration of a method that allows the preparation of populations of RPE-1 cells synchronized by enhanced mitotic arrest. The authors present the convincing argument that appropriate cell cycle analytic methods are required for the increasingly important RPE- 1 cell, a model karyotypically normal, untransformed epithelial cell type. A serial G1 and mitotic block, accomplished with palbociclib and nocodazole, respectively, resulted in populations with 80-90% of cells arrested in a prometaphase-like state, which proceeded synchronously to exit mitosis upon removal of nocodazole. The utility of the mitotically arrested population was demonstrated by immunoprecipitation of Ndc80 and associated proteins from extracts prepared from synchronized cells.

© 2020. Published by The Company of Biologists under the terms of the Creative Commons Attribution License (https://creativecommons.org/licenses/by/4.0/).

In summary, this is a highly focused report describing a novel method to produce populations of nearly pure mitotic RPE-1 cells. As RPE-1 and its derivatives are increasingly important models for cell biology, it is likely that this method will be of significant utility in the field.

Comments for the Author
Overall, this is a reasonably well documented description of a method that is likely to be of significant utility to labs using RPE-1 cells and derivatives to study mitosis and possibly cell cycle events. A general lack of quantitative information is seen as a limit to the utility of the method as presented. As well, the authors focused on the mitotic population and did not develop the utility of the method as a general tool for cell cycle studies. Nonetheless, the method seems sound and should be a substantive contribution to the establishment of robust methods to study the cell biology of RPE-1 cells.
We are pleased that the Reviewer found our methodology sound and useful. S/he raised a few comments, which we found to be thoughtful and helpful. Our point-by-point responses to the Reviewer's comments are below.

Specific comments are below:

1. While the method is clear in terms of cell manipulations, culture conditions and drug concentrations, the technique begins by splitting the cells 1/6. It would be very useful to know what cell density this corresponds to to allow quantitative planning of synchrony experiments. Similarly for the preparation of extract for immunoprecipitation - how many cells is 5 x 175 cm^2? Was the protein concentration of the extract determined.
We are sorry that some of our Methods were not described in sufficient detail. We have now included the information requested by the Reviewer at pages 7 and 9 of the revised version. We checked the quality and amount of proteins before and after immunoprecipitation by both Western blot (new Fig 4A) and silver staining, but unfortunately did not quantify the concentration of the total proteins in the extracts and therefore cannot offer this information to the Reviewer.

2. The authors show clearly that cells are arrested in a prometaphase-like state and removal of nocodazole results in prompt exit from mitosis. How does the population behave in the subsequent cell cycle? Is this a method that is applicable to general cell cycle studies? The authors should comment on this.
The aim of our methodology is to harvest large quantities of cells synchronized at different mitotic stages, but we understand the concern of the Reviewer and agree that information about how RPE-1 cells behave after the synchronization would be useful. To address this, we have extended our analysis and followed the survival and behavior of RPE-1 cells up to 48 hour after release from nocodazole. As shown in the new Figure 3, RPE-1 cells re-attach very rapidly - just 3 hours after nocodazole release - and reacquire a normal morphology after 24 hours. Furthermore, flow cytometry profiles indicated that almost all cells were diploid after 3 hours and then slowly re-entered the cell cycle to reacquire the normal profile of an unsynchronized cell population after 48 hours.

3. Ndc80 IP analysis reveals an unexpected enrichment in RNA splicing components. Recent results have shown mitotic roles for such proteins, e.g. Kim et al J Biol Chem. 2019 May 31;294(22):8760-8772 (cohesin associated), Pellacani et al Elife. 2018 Nov 26;7. pii: e40325. doi: 10.7554/eLife.40325 (Ndc80 associated). Since this is a pronounced outcome of the IP experiment, perhaps a bit more discussion of recent findings is in order.
We thank the Reviewer for pointing this out and for the helpful suggestions. We have discussed the implication of our results in the context of the studies indicated by the Reviewer in the revised version of the manuscript at page 7, lines 173-179.

Reviewer 2
Advance Summary and Potential Significance to Field
This manuscript does not make a conceptual advance but is rather intended as a significant advance in methodology. It is true that RPE-1 cells have become a widely used model for studying the cell cycle and mitosis in particular, mainly through microscopy. Efficient methods of cell cycle synchronisation are of value in order to use this cell line for biochemical analysis. While some have claimed ability to synchronize these cells at G1/S with thymidine, this may be inefficient.

© 2020. Published by The Company of Biologists under the terms of the Creative Commons Attribution License (https://creativecommons.org/licenses/by/4.0/).

Other methods reported for RPE- 1 cells are use of a CDK1 inhibitor (RO-3306) to block cells at the G2/M border before release into mitosis, and trapping mitotic cells with nocodazole without prior synchronisation, although this latter method obviously results in fewer mitotic cells. The present method of presynchronization in G1/S with the CDK4/6 PD 0332991 (palbociclib) followed by release and capture in mitosis with nocodazole is an addition to this methodology, albeit a minor advance in itself.

A concern (that is often neglected) with any drug-induced synchronisation method is the validity of the synchronised cells as a model for normal cell cycle states. Inevitably, synchronisation involves the induction of a stress response in the cells, which is why they halt cell cycle progression. It is important to know (1) are there persistent consequences for the cell after the drug treatment, (2) do the cells recover from the synchronisation to resume normal cell cycle processes. For instance, it is very likely that inhibition of DNA replication by thymidine synchronisation (and CDK4/6 inhibition?) causes replication stress, the effects of which may persist into a subsequent mitosis. In addition, it is clear that nocodazole arrest of cells in mitosis triggers a stress response that can even lead to cell death, depending on the duration of the arrest. Importantly, recovery from nocodazole arrest through repolymerization of microtubules and formation of the mitotic spindle is often inefficient, leading to defects in chromosome segregation and downstream consequences, including aneuploidy and interphase stress responses. It is therefore important to fully characterise both the stress response to CDK4/6 inhibitor synchronisation followed by nocodazole synchronisation, and to demonstrate that cells thus synchronised perform a (relatively) normal mitosis.

My view is that this work would be better published as part of a more comprehensive paper on the NDC80 complex, including conceptual advances, or as a well characterised study comparing synchronisation methods to demonstrate the benefits and limitations of each method.
Our initial intention was indeed to publish this method in a more comprehensive study. However, the Covid-19 pandemic has created great uncertainty and considerably hindered the progress of our research insofar that we have no idea of when we will be able to complete this study. As we reasoned that our methodology could be useful to the cell cycle community, we decided to publish it as a stand- alone method report. The considerable interest that our preprint received (https://www.biorxiv.org/content/10.1101/2020.04.21.052803v1) indicates that our decision was right.

Comments for the Author
To demonstrate that CDK4/6 inhibitor synchronisation followed by nocodazole synchronisation is a superior method of collecting cells to study mitosis, I suggest that the authors characterise the levels of key indicators of stress responses following release from each of these treatment, including DNA damage responses that would indicate whether or not the cells have replication stress or activation of mitotic stress responses.
Analysis of gamma H2AX levels would be a good place to start, preferably by microscopy but also by western blotting. The levels of cell death induced by the method will also be important to check. From the flow cytometry profiles shown, it does look like some cells with sub-2N DNA content are generated, which probably indicates apoptosis or possibly chromosome missegregation. At a low level, this might be acceptable, but it calls into question whether the synchronised cells truely have a normal mitosis and the stress response could affect mitotic events.
We understand the concern of the Reviewer, but would like to point out that several studies have already demonstrated that palbociclib does not cause DNA damage in several cell lines, including non- transformed epithelial cells (Dean et al., 2012; DiRocco et al., 2014; Hu et al., 2016; Roberts et al., 2012; now cited in the manuscript). Moreover, a preprint posted by Hagan's lab while our manuscript was under review reported that palbociclib does not cause DNA damage in RPE-1 cells (https://www.biorxiv.org/content/10.1101/2020.07.04.187625v1). Therefore, we think that it is unnecessary to repeat this analysis in our study, but have included this published information in the manuscript (page 4, lines 96-98).

The Reviewer is correct in pointing out that the <2*n* population indicates that some cell death occurs during synchronization, but this was very low (<5%), which clearly indicates that our synchronization method does not significantly affect cell viability. We have now included this information in the text (page 5, lines 115-118) and apologize for not making this clear in our previous version of the manuscript.

© 2020. Published by The Company of Biologists under the terms of the Creative Commons Attribution License (https://creativecommons.org/licenses/by/4.0/).

Secondly, it seems important to confirm that the synchronised cells perform mitotic correctly and on schedule in order to demonstrate that this is a good or improved technique for studying mitosis. This needs to be done by microscopy, preferably live cell imaging.

This comment of the Reviewer obviously applies to all synchronization methods that use nocodazole and not specifically to ours. The Reviewer would concur that the degradation of cyclin B observed after nocodazole release (Fig. 2) indicates that the spindle assembly checkpoint (SAC) had been satisfied and thus bipolar chromatid attachments had formed. Moreover, it has already been described that mitotic spindle assembly occurs after nocodazole washout in RPE-1 and other cell lines (Cimini et al., 2001; Worrall et al., 2018; now cited in the manuscript), although these studies reported an increase in the frequency of merotelic attachments (which are not sensed by the SAC) and chromosome mis- segregation. Therefore, we think that it is unnecessary to repeat these already published experiments especially considering that (i) time-lapse imaging of RPE-1 cells after mitotic shake off is extremely challenging and time consuming because cells are in suspension and (ii) our data also indicate that they exit mitosis very rapidly (within 60 minutes; Fig. 2). Furthermore, the University of Cambridge has only recently reopened at highly reduced capacity after the lockdown imposed because of the Covid-19 pandemic and we won't have access to the imaging facility, which is located in a different building, for at least another two months and this access will be very limited. Finally, we now include new evidence that all RPE-1 cells are in G1 three hours after nocodazole washout, reacquire their normal morphology after 24 hours, and re-enter the cell cycle after 48 hours (new Fig. 3), clearly indicating that our method does not significantly impair the survival and functionality of RPE-1 cells. However, we do share the Reviewer's concern that incorrect merotelic attachments and chromosome mis-segregation may occur after nocodazole release and have therefore commented on this at the end of the Discussion section (page 7, lines 180-186).

Second decision letter

MS ID#: JOCES/2020/247940

MS TITLE: Synchronization of human retinal pigment ephitilial-1 (RPE-1) cells in mitosis

AUTHORS: Stacey J Scott, Kethan S Suvarna, and Pier Paolo D'Avino
ARTICLE TYPE: Tools and Resources

I am happy to tell you that your manuscript has been accepted for publication in Journal of Cell Science, pending standard ethics checks.

Reviewer 1

Advance summary and potential significance to field

This paper presents a simple and effective pharmacological arrest-release protocol that provides highly synchronous populations of RPE-1 cells in mitosis. This method may be of significant utility to workers using RPE-1 and derivatives to study mitotic processes, particularly at a biochemical level.

Comments for the author

I feel that the modifications introduced after consideration of reviewer comments have improved the manuscript. I understand the other reviewer's point that this should be part of a larger study, but feel the authors' concern about conducting such a study under current conditions are legitimate. The result is a methodological paper that is limited in scope but very clear in execution and results. I do not feel that any further revision in the absence of new experiments will improve this manuscript.

© 2020. Published by The Company of Biologists under the terms of the Creative Commons Attribution License (https://creativecommons.org/licenses/by/4.0/).

Reviewer 2

Advance summary and potential significance to field

The paper provides evidence of the use of an improved method of cell cycle synchronisation which will be of utility in the biochemical analysis of mitosis.

Comments for the author

The authors have made some modifications to the manuscript which are helpful. The inclusion of comparative data showing the advantage of the synchronisation methodology and lack of DNA damage or chromosome defects in mitosis would have greatly strengthened the paper. It is obviously not a perfect study but they are clearly constrained at present with regard to providing new data. However, on balance, the work probably justifies publication.

© 2020. Published by The Company of Biologists under the terms of the Creative Commons Attribution License (https://creativecommons.org/licenses/by/4.0/).
